# Accumulation of free cholesterol and oxidized low-density lipoprotein is associated with portal inflammation and fibrosis in nonalcoholic fatty liver disease

**DOI:** 10.1186/s12950-019-0211-5

**Published:** 2019-04-02

**Authors:** Cheng-Maw Ho, Shu-Li Ho, Yung-Ming Jeng, Yu-Sheng Lai, Ya-Hui Chen, Shao-Chun Lu, Hui-Ling Chen, Po-Yuan Chang, Rey-Heng Hu, Po-Huang Lee

**Affiliations:** 1 Department of Surgery, National Taiwan University Hospital and College of Medicine, Taipei, Taiwan; 2 Hepatitis Research Center, National Taiwan University Hospital, 7 Chung-Shan South Road, Taipei, 100 Taiwan; 3 Department of Pathology, National Taiwan University Hospital and College of Medicine, Taipei, Taiwan; 4 Department of Biochemistry and Molecular Biology, National Taiwan University College of Medicine, 1, Jen Ai Rd, Sec 1, Taipei, 100 Taiwan; 5 Department of Pediatrics, National Taiwan University Children Hospital, Taipei, Taiwan; 6 Cardiovascular Center and Division of Cardiology, Department of Internal Medicine, National Taiwan University Hospital and College of Medicine, Taipei, Taiwan; 7 Department of Surgery, E-Da Hospital, I-Shou University, Kaohsiung, Taiwan; 8 Department of Anatomy and Cell Biology, National Yang-Ming University, Taipei, Taiwan

**Keywords:** Nonalcoholic fatty liver disease, Steatohepatitis, Oxidized low-density lipoprotein

## Abstract

**Background:**

Macrophages engulf oxidized-LDL (oxLDL) leading to accumulation of cellular cholesterol and formation of foam cells, which is a hallmark of atherosclerosis. Moreover, recent studies showed that accumulation of free cholesterol in macrophages leading to activation of NLRP3 inflammasome and production of interleukin-1β (IL-1β) has been linked to atherosclerosis-associated inflammation. However, it is not clear if cholesterol accumulation is associated with hepatic inflammation and fibrosis in the liver. In this study, we investigated the association of free cholesterol and oxLDL accumulation in portal vein with the inflammation, atherosclerosis, and fibrosis in human nonalcoholic fatty liver disease (NAFLD).

**Methods:**

Serial sections derived from surgical specimens of NAFLD were stained with filipin and antibodies against IL-1β, CD68, α-smooth muscle actin (α-SMA), oxLDL and lectin-like oxLDL receptor-1 (LOX-1).

**Results:**

We show that free cholesterol was colocalized with oxLDL in the wall of portal vein, and which was associated with lumen narrowing, plaque formation, endothelium deformation, and portal venous inflammation. The inflammation was evidenced by the colocalization of Kupffer cells and IL-1β and the expression of LOX-1. Notably, ruptured plaque was closely associated with portal venous inflammation. Moreover, free cholesterol and oxLDL accumulation in periportal and sinusoidal fibrosis, which was associated with regional stellate cell activation and chicken-wire fibrosis.

**Conclusion:**

These findings reveal a direct association between cholesterol accumulation, portal venous inflammation and fibrosis in NAFLD.

**Electronic supplementary material:**

The online version of this article (10.1186/s12950-019-0211-5) contains supplementary material, which is available to authorized users.

## Introduction

Nonalcoholic fatty liver disease (NAFLD) encompasses a wide spectrum of progressive liver diseases, namely macrovesicular fat accumulation (simple steatosis), nonalcoholic steatohepatitis (NASH), fibrosis, and cirrhosis. Simple steatosis is considered a benign condition, whereas the other conditions are often considered progressive, and they have been increasingly recognized as important risk factors for hepatocellular carcinoma and as conditions that warrant liver transplantation [1, 2]. Although significant data have been obtained to support the “two-hit hypothesis”, important molecular mechanisms underlying NAFLD progression remain elusive [3].

NAFLD is the hepatic manifestation of metabolic syndrome [3, 4]. Patients with NAFLD have a higher risk of cardiovascular mortality than those without NAFLD [5]. Chronic low-grade inflammation mediated by macrophages is a common factor involved in the pathogenesis of a collection of metabolic pathologies, including insulin resistance, NAFLD, atherosclerosis, and dyslipidemia [6–8]. Specifically, macrophage activation mediated by lipid metabolites, such as oxidized lipoproteins, free fatty acids, free (unesterified) cholesterol, and ceramides, represents a common pathogenic lipotoxic mechanism [9].

Oxidized low-density lipoprotein (oxLDL) is a key pathogenic determinant of vascular atherosclerosis. Moreover, increased levels of oxLDL are associated with an increased incidence of metabolic syndrome [10], acute coronary events [11], and hepatocellular injury in experimental cholestasis and fibrosis [12]. The short-term administration of oxLDL to high-fat diet-fed mice not only aggravated hepatic steatosis but also resulted in inflammatory cell infiltration and subsequent severe hepatic injury, which are typical histological features of NASH [13]. OxLDL has been established to promote the production of inflammatory cytokines and chemotactic factors by macrophages [14] and to stimulate coronary artery smooth muscle cells to overexpress interleukin (IL)-1 [15], a crucial gatekeeper of inflammation and tissue damage [16].

Bieghs et al. demonstrated that, after entering Kupffer cells, oxLDL is trapped inside lysosomes, resulting in the increased expression of inflammatory genes, such as those for tumor necrosis factor (TNF)-α and toll-like receptor (TLR)-4 [17]. Collective evidence suggests that the unregulated uptake of oxLDL by scavenger receptors in Kupffer cells leads to the accumulation of lysosomal free cholesterol (oxLDL-derived lipid metabolites) and triggers an inflammatory response in experimental NASH [18]. We found the accumulation of free cholesterol in the periportal area and detected the colocalization of enlarged Kupffer cells and electronegative LDL (mildly oxidized LDL found in blood) in the portal venous area of high-fat, high-cholesterol (HFC) diet-fed hamsters [19]. These factors were found to be associated with the development of NASH and fibrosis [19]. Moreover, we demonstrated that electronegative LDL, which was isolated from the blood of HFC diet-fed hamsters, induced TNF-α production in rat Kupffer cells through a lectin-like oxLDL receptor (LOX)-1 (a scavenger receptor)-dependent pathway [19]. These results suggest a causal role of electronegative LDL and LOX-1 in the development of NASH. Moreover, the accumulation of free cholesterol can result in cholesterol crystallization in vessel walls. Cholesterol crystals have been shown to induce NLRP3 inflammasome activation and subsequent IL-1β production [20]. However, oxLDL particles, which accumulate in early atherosclerotic lesions, are a prerequisite for NLRP3 inflammasome-mediated IL-1β secretion [21]. Although these key mechanisms of oxLDL and free cholesterol-induced liver toxicity have been described in animal models, their relevance in human NASH remains unclear [18]. In a lipidomic analysis, Puri et al. reported a significant increase in hepatic free cholesterol in human NASH [22], but detailed hepatic distribution remains unknown. According to our review of relevant literature, no study has directly demonstrated both oxLDL and free cholesterol in human liver histology. Thus, the present study investigated the accumulation of oxLDL and free cholesterol in the hepatic microenvironment of NAFLD.

## Methods

### Surgical specimen preparation

The study was approved by the Institutional Review Board of National Taiwan University Hospital (NTUH REC: 201401050RINB). Residual liver tissues were obtained from surgical specimens (nontumor livers through partial hepatectomy or explants from liver transplantation) after obtaining informed consent from all patients or their guardians, if required. There was no evidence of excessive alcohol consumption or hepatitis B or C virus infection in the patients. The tissues were fixed in formalin, embedded in paraffin, sectioned, and stained with hematoxylin and eosin for hepatic histological examination. Snap-frozen tissue samples were analyzed using immunohistochemistry.

### NAFLD definition

NAFLD was diagnosed based on the evidence of hepatic steatosis from either imaging or histological results, only if hepatic steatosis was not caused by excessive alcohol consumption, steatogenic medication use, or hereditary disorders [23]. The liver specimen was considered to represent fatty liver disease if macrovesicular steatosis or steatohepatitis was predominant. Steatohepatitis was defined by the minimal criteria for hepatic steatosis, in addition to scattered, mainly lobular inflammation with or without Mallory bodies, cytologic ballooning, and perisinusoidal fibrosis [24].

### Immunofluorohistochemistry

Staining with antibodies against oxLDL, α-smooth muscle actin (SMA), LOX-1, CD68, CD11b, IL-1β, and CD31 was performed according to the manufacturers’ recommendations, as detailed in Additional file 1: Table S1.

Secondary antibodies, namely Alexa Fluor 488 donkey anti-rabbit immunoglobulin (IgG; Molecular Probes, Oregon, USA), Alexa Fluor 594 donkey anti-goat IgG, and Alexa Fluor 594 donkey anti-rat IgG, were used in immunofluorescence assays. Nuclei were stained with 4′,6-diamidino-2-phenylindole (DAPI) or propidium iodide. Moreover, images were obtained using digital cameras (DP21, Olympus, Japan or SPOT™ Imaging Solutions, Diagnostic Instruments, Inc., Michigan, USA).

### Histological evaluation

Immunostaining of the hepatic microenvironment was evaluated and compared in serial sections. Liver specimens from a recipient with propionic acidemia and those from a healthy living donor were used as controls. α-SMA-positive cells were considered as vascular smooth muscle cells if they were located in the vascular walls or as activated stellate cells if they were located elsewhere and without CD31 on the adjacent endothelial cell surface. CD68-positive cells were considered as macrophages. LOX-1 has been established to be expressed in vascular endothelial and smooth muscle cells and implicated in endothelial dysfunction and atherosclerosis [25, 26]. Moreover, it is expressed in activated macrophages in the proinflammatory microenviroment [27–29]. Irregularly stained expression pattern of vessels suggested vascular damage [30]. In addition to the expression of IL-1β, the induced expression of LOX-1 beyond that in the control tissue was considered a sign of inflammation [30]. Fibrosis was validated through Masson’s trichrome staining. Free cholesterol was identified through filipin staining. At least 5 portal venous regions were examined. Liver tissues from a pediatric patient with propionic acidemia (Additional file 2: Figure S1A) and those from a living adult donor (Additional file 2: Figure S1B) with a pathological diagnosis of mild fatty liver (5%) were used as the comparative control.

## Results

### Histopathology of human NAFLD

**Fig. 1:**
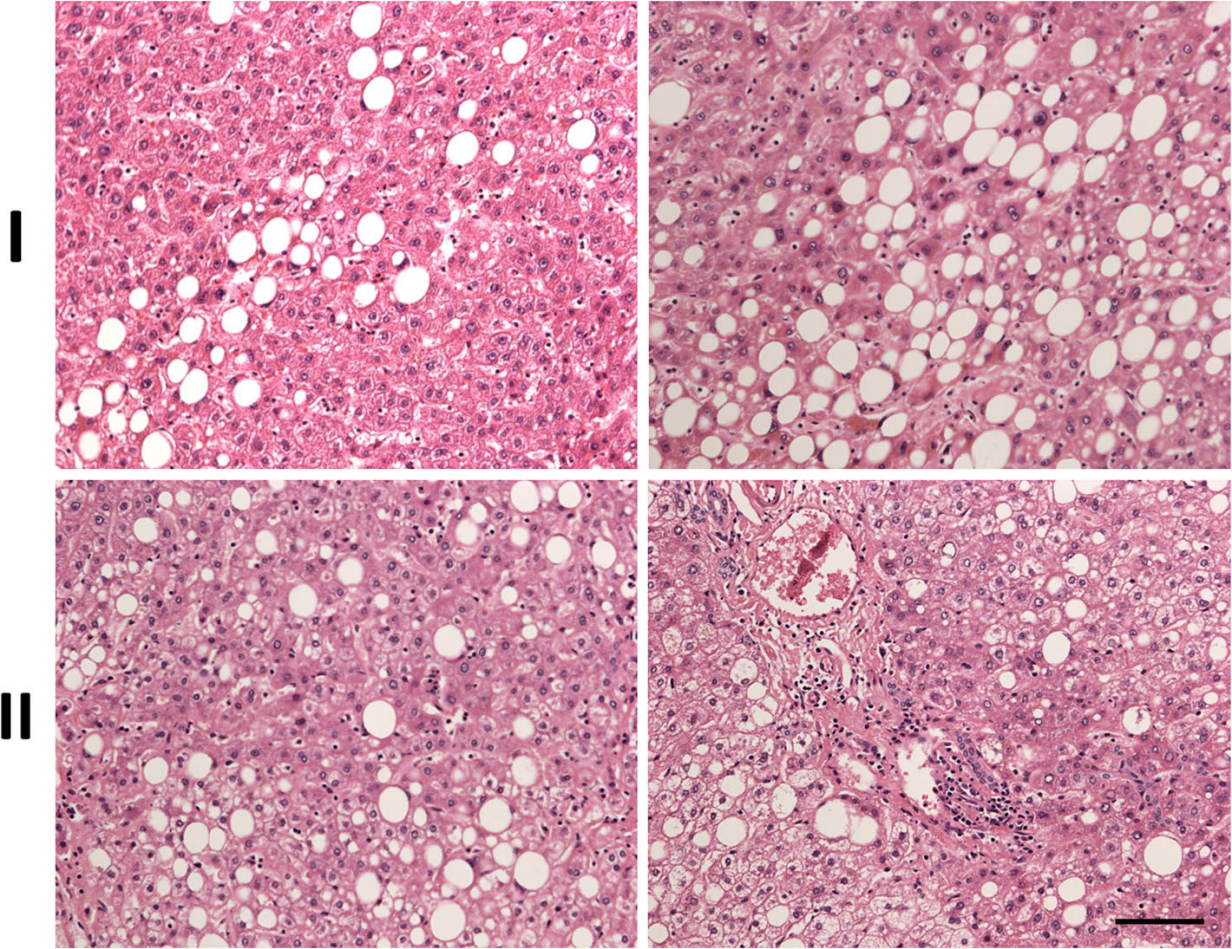
Histopathology of human NAFLD. Two surgical specimens (I and II) were stained with hematoxylin and eosin (H&E). Macrovesicular steatosis and portal venous inflammation are observed in the hepatic lobule. Scale bar: 100 μm

Histopathology of two surgical specimens (I and II) for further analysis were shown in Fig. 1. Specimen I, from non-tumor liver part of an 82-year-old man with a grade II hepatocellular carcinoma, revealed 10–20% macrovesicular fatty change. Hepatitis and fibrosis (Metavir score 1) were also noted. Specimen II, from non-tumor liver part of a 75-year-old man with combined hepatocellular- and cholangio-carcinoma, showed 30% fatty change and heterogeneous active cirrhosis.

### Focal narrowing of intrahepatic portal vein was associated with oxLDL and free cholesterol-rich plaque formation

**Fig. 2:**
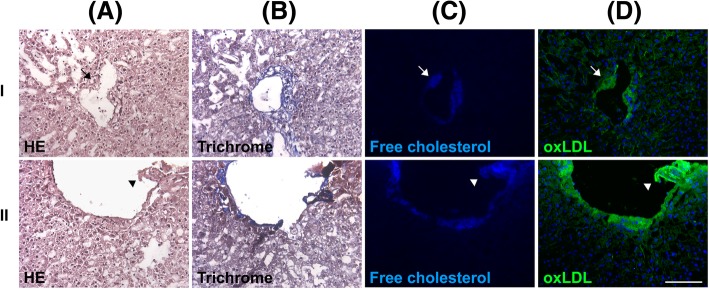
Portal venous atherosclerosis in human NAFLD. Two surgical specimens (I and II) were stained with hematoxylin and eosin (H&E) (**a**), trichrome (**b**), filipin (**c**), or an antibody against oxLDL (**d**). The portal venous plaque showed oxLDL and free cholesterol accumulation. The plaque caused the narrowing of the portal venous lumen (arrows, I) or was protruding and thus ruptured the vein (arrowheads, II). Scale bar: 100 μm

**Fig. 3:**
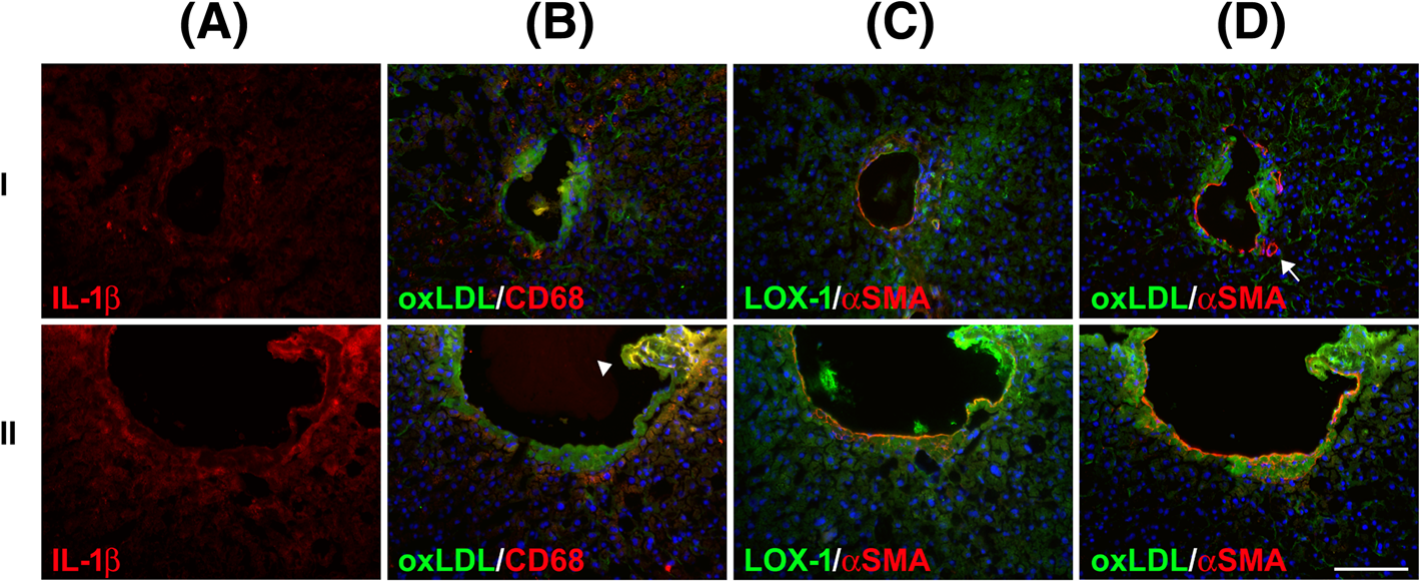
Portal venous plaque inflammation marked by macrophage infiltration in NAFLD. The surgical specimens (I and II) were stained with antibodies against IL-1β (**a**), oxLDL and CD68 (**b**), LOX-1 and α-SMA (**c**), and oxLDL and α-SMA (**d**). Macrophage infiltration (marked by CD68) in the portal vein walls with oxLDL accumulation is shown. A ruptured portal venous plaque showed a particularly high intensity of CD68, LOX-1, and IL-1β signals, suggesting portal venous inflammation (lI, arrowhead). Portal venous atherosclerosis was observed in the layer below vascular smooth muscle (marked by α-SMA) and protruded into the portal venous lumen. The hepatic artery (arrow) was not involved in the oxLDL-related event. Scale bar: 100 μm

Histologically, the focal narrowing of the intrahepatic portal vein with plaque formation was frequently observed (Fig. 2). The plaque showed free cholesterol and oxLDL accumulation in the same region (Fig. 2, arrows and arrowheads, respectively). The closely associated colocalization suggested that the free cholesterol was derived from oxLDL. The pattern of appearance was similar to that for arterial atherosclerosis in coronary arterial disease [31]; however, this phenomenon was not observed in the intrahepatic artery (Fig. 3, arrow, and Additional file 3: Figure S2, arrowhead, upper panel). The involved endothelium became thickened and morphologically deformed because of chronic hepatitis (Additional file 3: Figure S2, lower panel). However, oxLDL accumulation was not very frequently observed in the nearby parenchyma where fibrosis was absent.

### oxLDL-rich portal venous plaque was associated with IL-1β, Kupffer cell, and LOX-1 overexpression

The accumulation of free cholesterol results in the formation of cholesterol crystals, which can induce NLRP3 inflammasome activation [32] and subsequent IL-1β production [33]. The levels of IL-1β were high in the region with free cholesterol and oxLDL accumulation (Fig. 3a, lower panel). Furthermore, the colocalization of CD68-positive macrophages was observed (Fig. 3b, lower panel). The close association among free cholesterol, oxLDL macrophages, and IL-1β strongly support that cholesterol crystals are metabolic signals that trigger inflammation in atherosclerosis and NAFLD. In a previous animal study, we established an association of LOX-1-mediated inflammation and NASH [19]. We subsequently determined the association between LOX-1 and portal venous inflammation in human NASH. Figure 3c revealed that the ruptured portal venous plaque showed LOX-1 overexpression (Fig. 3c, lower panel). Similar to atherosclerosis, oxLDL was accumulated in the subendothelial layer, as revealed by α-SMA staining (Fig. 3d). Macrophages in the oxLDL-rich plaque were associated with endothelial injury and subsequent endothelium deformity (Figs. 3d and Additional file 3: Figure S2, arrowhead, lower panel). Specifically, the continuity of α-SMA staining integrity was interrupted in fragile regions of the oxLDL-rich plaque, with endothelial disruption (Figs. 3d and Additional file 3: Figure S2, lower panel). Taken together with our previous animal analysis, we established the association of oxLDL, macrophages, and IL-1β (Fig. 3) with LOX-1-mediated inflammation [19].

To delineate whether macrophages originate from bloodstream monocytes, which express CD11b [34] in fragile plaques, additional characterization was performed. The results suggested that Kupffer cells were the major macrophages (Additional file 4: Figure S3).

### oxLDL accumulation in cirrhotic liver parenchyma

**Fig. 4:**
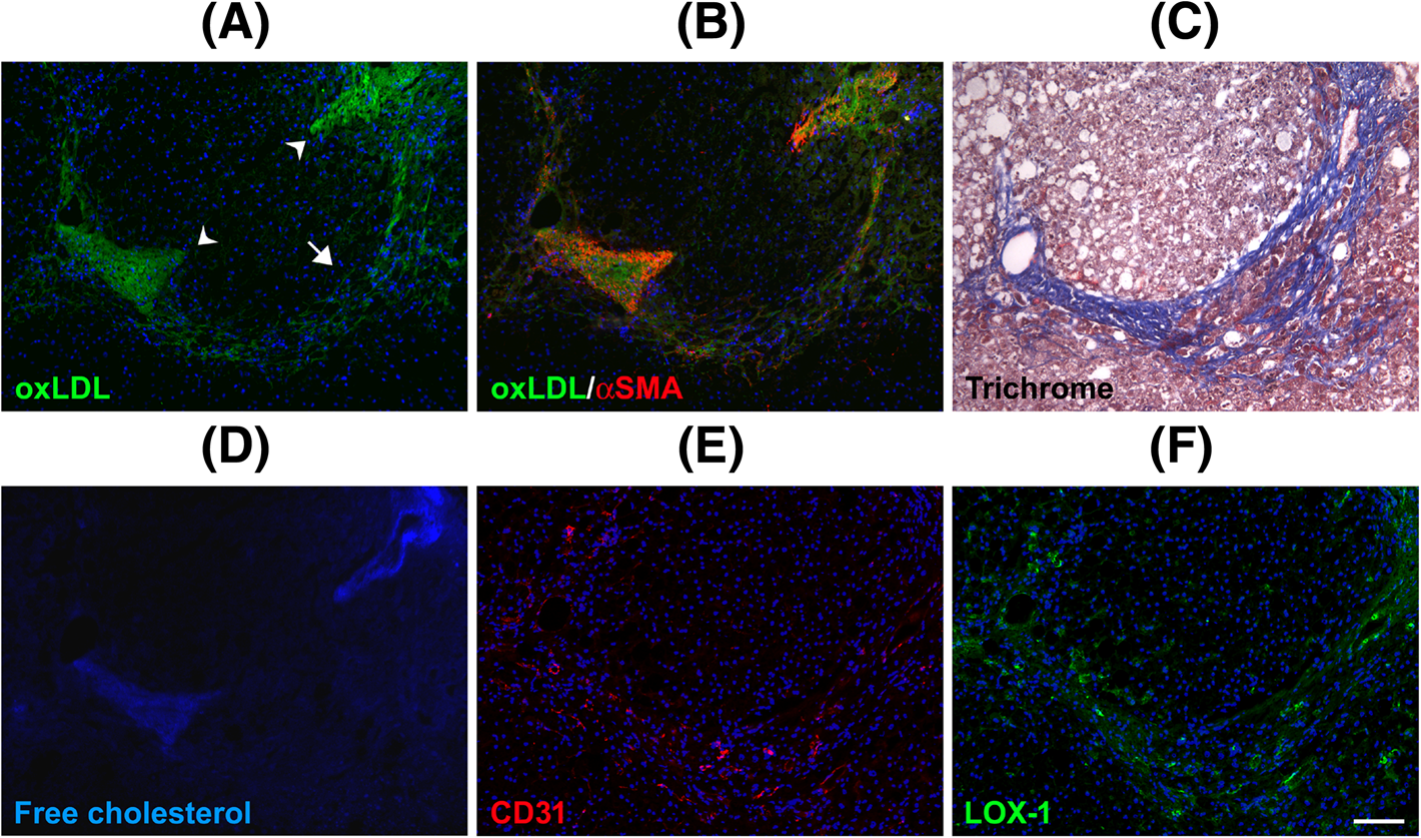
Association of free cholesterol and oxLDL with chicken-wire fibrosis in NAFLD. The surgical specimens were stained with an antibody against oxLDL (**a**), antibodies against oxLDL and αSMA (**b**), trichrome (**c**), filipin (**d**), an antibody against CD31 (**e**), and an antibody against LOX-1 (**f**). Chicken-wire fibrosis was observed through trichrome staining. oxLDL and free cholesterol were observed in the fibrotic parenchyma of cirrhosis (arrow) and were also associated with each other. Activated stellate cells (marked by αSMA without CD31 in the surrounding environment) infiltrated the fibrotic parenchyma, where oxLDL and free cholesterol were accumulated. The phenomenon was prominent adjacent to the portal vein (arrowheads). LOX-1 expression in the fibrotic parenchyma was not high and was not closely related to oxLDL accumulation. **a** high-power field of magnification is shown in Additional file 4: Figure S3. Scale bar: 100 μm

oxLDL accumulation was not observed in most areas of the sinusoidal parenchyma in noncirrhotic livers. This finding was possibly because most scavenger receptors are located in the sinusoidal endothelium. oxLDL accumulation was prominent from the portal vein outward to the sinusoidal parenchyma in cirrhotic livers, accounting for the chicken-wire appearance (Fig. 4). The association between fibrosis and chicken-wire appearance was determined through trichrome staining (Fig. 4). Notably, in the sinusoidal parenchyma, oxLDL was strongly correlated with free cholesterol (Fig. 4). However, macrophages were not active in these areas (Additional file 5: Figure S4), and activated stellate cells (marked by α-SMA and not related to CD31) were prominent, particularly in areas of free cholesterol accumulation (Fig. 4). Recent evidence suggests that free cholesterol accumulation in hepatic stellate cells directly activates these cells, possibly through a TLR-4-dependent pathway [18]. LOX-1 expression in the sinusoidal parenchyma was low (Additional file 6: Figure S5). Hu et al. reported that collagen synthesis in mouse cardiac fibroblasts involves a facilitative interaction between tumor growth factor-β1–NADPH oxidase and LOX-1 [35]. However, the association of oxLDL with stellate cell activation fibrosis observed in this study seemed independent of LOX-1.

Demographics of patients who provided source livers were detailed in Additional file 7: Supplemental results.

## Discussion

**Fig. 5:**
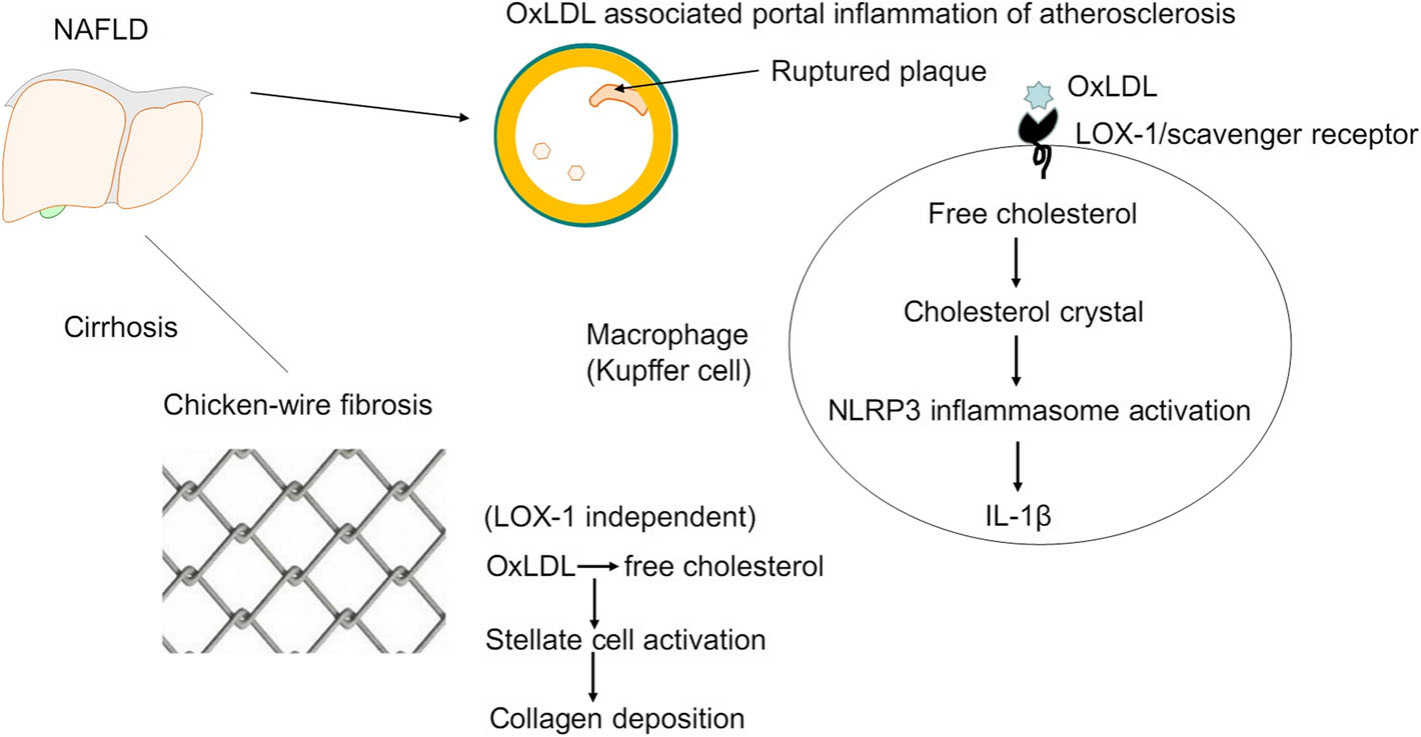
Schematic illustration of oxLDL accumulation in human NAFLD. oxLDL accumulation in the portal vein wall resulted in lumen narrowing, plaque formation, and LOX-1-mediated inflammation, particularly in fragile plaques. oxLDL and free cholesterol accumulation in cirrhotic parenchyma was associated with chicken-wire fibrosis through stellate cell activation. Notably, free cholesterol accumulation in the vessel wall can form crystals, which induce NLRP3 inflammasome activation and subsequent IL-1β production [20, 21]. This activity may destabilize atherosclerotic plaques and account for plaque rupture [32]

The present study revealed that free cholesterol and oxLDL accumulation in the portal venous wall, instead of the hepatic artery, caused focal narrowing of the intrahepatic portal vein by inducing portal venous atherosclerosis in NAFLD. Ruptured portal venous plaques were closely associated with the local expression of free cholesterol, oxLDL, LOX-1, IL-1β, and Kupffer cells. Free cholesterol and oxLDL accumulation in periportal and sinusoidal fibrosis, which was associated with regional stellate cell activation and chicken-wire fibrosis. Figure 5 presents a graphic summary of the tentative rationale. Our study demonstrated that free cholesterol and oxLDL play specific roles in the pathogenesis of NAFLD. These initial results bridge the knowledge gap between atherosclerosis and NASH in metabolic syndrome and suggest that free cholesterol and oxLDL are the missing link and deserve translational investigation in humans. Moreover, our results implied that additional studies should emphasize atherosclerosis in the portal vein, rather than in the hepatic artery, when addressing oxLDL-related vascular injury in NAFLD.

Consistent with the results of our previous animal study [19], the current histopathological observation in human NAFLD showed an association between LOX-1 and Kupffer cell-associated inflammation, which was closely attributed to oxLDL. In our animal model of NASH, active inflammation was documented in the liver parenchyma [19]. Compared with the previous animal model study, in the current study, human surgical specimens of NAFLD showed low-grade parenchymal inflammation but active inflammation at focal fragile portal venous plaques. The phenotypic difference may be explained by the longer duration and lower dose of exposure of oxLDL and free cholesterol in humans than in animals. Furthermore, the specimens were selectively sampled from patients having a low inflammatory status of the liver who were scheduled to undergo partial hepatectomy. Selection bias cannot be avoided. However, the findings revealed that portal venous atherosclerosis, which is novel, occurred in actual clinical scenarios and warrant corresponding studies. Consistently, a previous study reported that increased periportal chronic inflammation is associated with the multiple clinical and pathological features of progressive NAFLD [36]. Altogether, these findings suggest that portal venous atherosclerosis and inflammation in NAFLD are attributed to free cholesterol and oxLDL accumulation. However, additional studies are warranted to consolidate our understanding of the phenomenon.

We found several LOX-1-containing oxLDL-rich fragments with activated macrophages in the portal venous lumen. These particles may be cleaved by enhanced protease activity locally [37] and separated from fragile portal venous plaques, as seen in coronary atherosclerosis concurrent with cardiovascular accidents. Moreover, a previous study reported that the level of soluble LOX-1 is increased in NAFLD patients with steatohepatitis [38]. It remains unknown whether the separated fragments of portal venous plaques cause distal downstream liver damage, thus warranting further investigation.

In the cirrhotic stage of NAFLD, oxLDL presents a chicken-wire pattern in addition to activated stellate cells and fibrosis. LOX-1 expression was not obvious in this stage. Liver sinusoidal endothelial cells and Kupffer cells constitute the largest scavenger cell system for oxLDL in the body [39]. They can effectively eliminate oxLDL in noncirrhotic livers, which might explain the rarity of oxLDL particles in the noncirrhotic sinusoidal parenchyma. Therefore, the accumulation of oxLDL and free cholesterol in the cirrhotic parenchyma suggests the dysfunction of the hepatic scavenger system. Liver fibrosis involves complex dynamic interactions among stellate cells, sinusoidal endothelial cells, Kupffer cells, hepatocytes, and cholangiocytes [40]. oxLDL can induce the activation of stellate cells by increasing the expression of α(I) collagen; α-SMA; and profibrogenic genes, such as those for connective tissue growth factor and transforming growth factor-β receptors in vitro [41]. Furthermore, the accumulation of free cholesterol in cells can cause the progression of liver fibrosis through stellate cells activation by disrupting the integrity of mitochondrial and endoplasmic reticulum (ER) membranes and by triggering mitochondrial oxidative injury and ER stress [42]. Altogether, patients with NAFLD can develop liver fibrosis through alternative pathways other than those mediated by LOX-1.

The limitation of this study is the potential coexistence of native LDL and oxLDL. However, the coexistence of native LDL and oxLDL would not be substantial because the extensively coaccumulated free cholesterol was not likely to have originated from native LDL [42]. The possible presence of other modified forms of LDL (e.g., aggregated LDL or LDL-containing immune complexes), which can also contribute to atherogenesis [43], was not excluded in this study. However, our previous study revealed that periportal oxLDL contributed to the development of experimental NASH, and that anti-LOX-1 agent blocked TNF-α production by Kupffer cells [19]. CD36 is another scavenger receptor for oxLDL and is associated with NLRP3 inflammasomes in NAFLD [7, 21]. We cannot exclude the possibility that and degrees to which IL-1β was contributed to by CD36. Besides, LOX-1 is a non-specific multiligand receptor sensing multiple danger signals, including oxLDL, C-reactive protein and C1q [26]. However, we qualitatively identified the involvement of LOX-1 and Kupffer cells in portal venous plaque inflammation. This is a novel finding in humans and support our observation in the experimental NASH model [19].

## Conclusions

Portal venous atherosclerosis induced by free cholesterol and oxLDL accumulation is a unique feature of NAFLD. A ruptured portal venous plaque closely associates oxLDL, LOX-1, and Kupffer cells with portal venous inflammation. In the cirrhotic stage, free cholesterol and oxLDL are associated with chicken-wire fibrosis through stellate cell activation. The present findings reveal a direct association between cholesterol accumulation, portal venous inflammation and fibrosis in NAFLD.

## Additional files

Additional file 1: **Table S1.** List of antibodies used in fluorescent immunohistochemistry. (DOC 36 kb)

Additional file 2: **Figure S1.** Control. (I). Panel staining of the liver explant specimen from a 1-year-5-month-old boy with propionic academia who underwent living donor liver transplantation. (II). Panel staining of the specimen from a 28-year-old man who donated his liver to his daughter and was histologically diagnosed with 5–10% fatty liver. Specimens were stained with H&E (A), trichrome (B), filipin (C), antibodies against oxLDL (D), IL-1β (E), oxLDL and CD68 (F), oxLDL and α–SMA (G), and α–SMA and LOX-1 (H) Nuclei were stained with DAPI (blue). Scale bar: 100 μm. (TIF 10907 kb)

Additional file 3: **Figure S2.** Destabilized portal venous atherosclerotic plaques and inflammation. oxLDL accumulation within the portal vein wall instead of the hepatic artery (arrowhead). oxLDL (+) particles were observed in the portal venous lumen (arrow). oxLDL accumulation induced macrophage infiltration (marked by CD68) and resulted in vessel wall deformity (lower panel). The strong colocalization of oxLDL and CD68 is shown in yellow (arrowhead, lower panel). Scale bar: 100 μm. (TIF 5744 kb)

Additional file 4: **Figure S3.** Macrophages in destabilizing atherosclerotic portal venous plaques. IL-1β-related macrophages (marked by CD68) were mainly CD11b (−), suggesting an origin from Kupffer cells and not from bloodstream monocytes [CD11b (+)]. Scale bar: 100 μm. (TIF 3657 kb)

Additional file 5: **Figure S4.** Inactive role of macrophages in fibrotic parenchyma in NAFLD. In the cirrhotic stage, oxLDL was observed in the fibrotic parenchyma. Macrophages (marked by CD68) were rarely visible. IL-1β expression was also low. Scale bar: 100 μm. (TIF 6498 kb)

Additional file 6: **Figure S5.** High-power field view of chicken-wire fibrosis in the cirrhotic stage of NAFLD. oxLDL accumulation in chicken-wire fibrosis in the parenchyma distal (a) and adjacent (b) to the portal vein. LOX-1 expression was low in the fibrotic parenchyma and was not closely related to oxLDL accumulation. Scale bar: 100 μm. (TIF 7340 kb)

Additional file 7: Supplemental Results. (DOCX 15 kb)

## Abbreviations

DAPI: 4′,6-diamidino-2-phenylindole
ER: Endoplasmic reticulum
HFC: High-fat, high-cholesterol
IL-1: Interleukin-1
LDL: Low-density lipoprotein
LOX-1: Lectin-like oxidized low-density lipoprotein receptor-1
NAFLD: Nonalcoholic fatty liver disease
NASH: Nonalcoholic steatohepatitis
OxLDL: Oxidized low-density lipoprotein
TLR-4: Toll-like receptor 4
TNF-α: Tumor necrosis factor-α
α-SMA: Alpha smooth muscle actin
