# Supplementary material for: Accumulation of free cholesterol and oxidized low-density lipoprotein is associated with portal inflammation and fibrosis in nonalcoholic fatty liver disease
Source: J Inflamm (Lond). 2019 Apr 2;16:7. doi: 10.1186/s12950-019-0211-5 (PMC6444889; doi:10.1186/s12950-019-0211-5)
Supplement: Supplementary file 1 — Table S1. List of antibodies used in fluorescent immunohistochemistry. (DOC 36 kb) [file 12950_2019_211_MOESM1_ESM.doc]

| **Supplemental Table S1 List of antibodies used in fluorescent immunohistochemistry** | | |  |  |
| --- | --- | --- | --- | --- |
|  |  |  |  |  |
|  | Name | Company | Cat. No. | Dilution |
| 1^st^ Antibody | Apo B-100 | Academy Biomedical Co., Houston, TX | 20A-G1b | 1:800 |
|  | CD11b | Biolegend, San Diego, CA | 301302 | 1:100 |
|  | CD31 | Biolegend, San Diego, CA | 303102 | 1:100 |
|  | CD68 | Serotec, Oxford, UK | MCA1957 | 1:200 |
|  | CD68 | Dako, Glostrup, Denmark | IS613 | Ready to use |
|  | Interleukin-1β ; IL-1β | abcam, Cambridge, UK | ab9722 | 1:150 |
|  | LOX-1 | abcam, Cambridge, UK | ab60178 | 1:150 |
|  | oxLDL | Millipore, Temecula, CA | AB3230 | 1:400 |
|  | αSMA | Dako, Glostrup, Denmark | M0851 | 1:400 |
| Others | Filipin III from Streptomyces filipinensis | Sigma, St Louis, MO | F9765 | 0.25mg/ml^＊^ |
| ^＊^Filipin was dissolved in dimethylsulfoxide then diluted to 0.25mg/ml in 10% FBS/PBS. | | |  |  |
