# Supplementary figures and images for: Accumulation of free cholesterol and oxidized low-density lipoprotein is associated with portal inflammation and fibrosis in nonalcoholic fatty liver disease
Source: J Inflamm (Lond). 2019 Apr 2;16:7. doi: 10.1186/s12950-019-0211-5 (PMC6444889; doi:10.1186/s12950-019-0211-5)

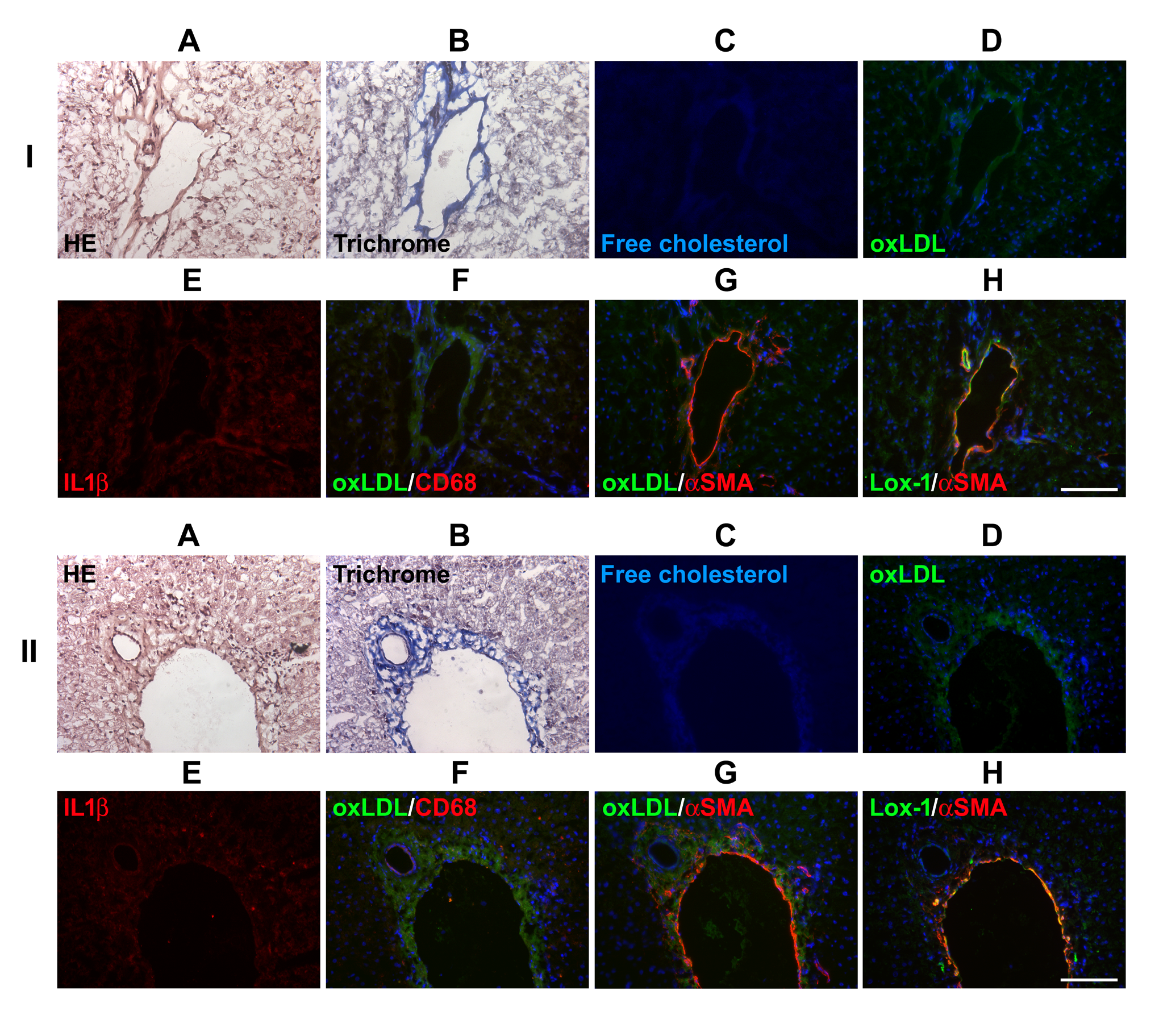

Supplement: Supplementary file 2 — Figure S1. Control. (I). Panel staining of the liver explant specimen from a 1-year-5-month-old boy with propionic academia who underwent living donor liver transplantation. (II). Panel staining of the specimen from a 28-year-old man who donated his liver to his daughter and was histologically diagnosed with 5–10% fatty liver. Specimens were stained with H&E (A), trichrome (B), filipin (C), antibodies against oxLDL (D), IL-1β (E), oxLDL and CD68 (F), oxLDL and α–SMA (G), and α–SMA and LOX-1 (H) Nuclei were stained with DAPI (blue). Scale bar: 100 μm. (TIF 10907 kb) [file 12950_2019_211_MOESM2_ESM.tif]

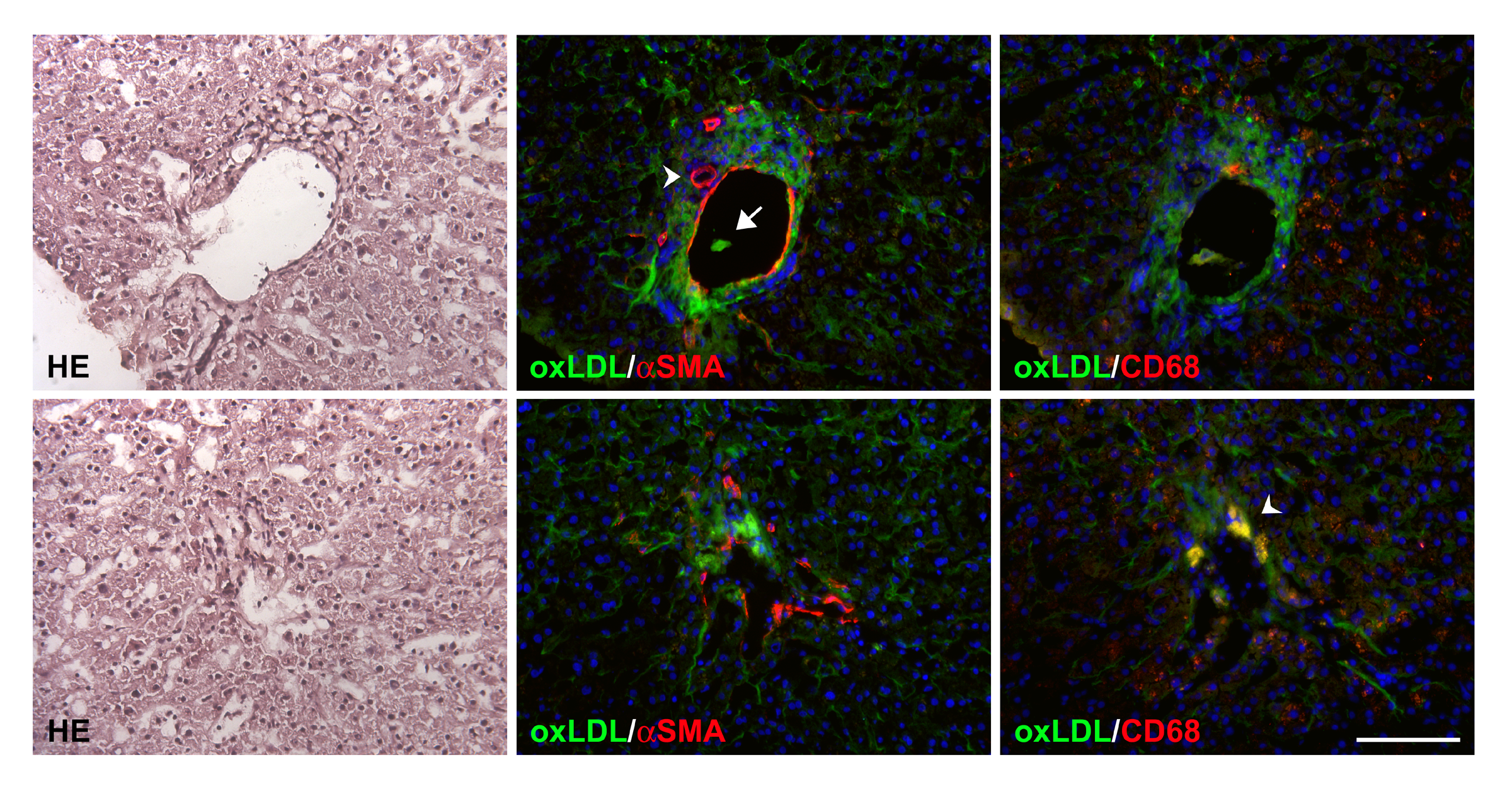

Supplement: Supplementary file 3 — Figure S2. Destabilized portal venous atherosclerotic plaques and inflammation. oxLDL accumulation within the portal vein wall instead of the hepatic artery (arrowhead). oxLDL (+) particles were observed in the portal venous lumen (arrow). oxLDL accumulation induced macrophage infiltration (marked by CD68) and resulted in vessel wall deformity (lower panel). The strong colocalization of oxLDL and CD68 is shown in yellow (arrowhead, lower panel). Scale bar: 100 μm. (TIF 5744 kb) [file 12950_2019_211_MOESM3_ESM.tif]

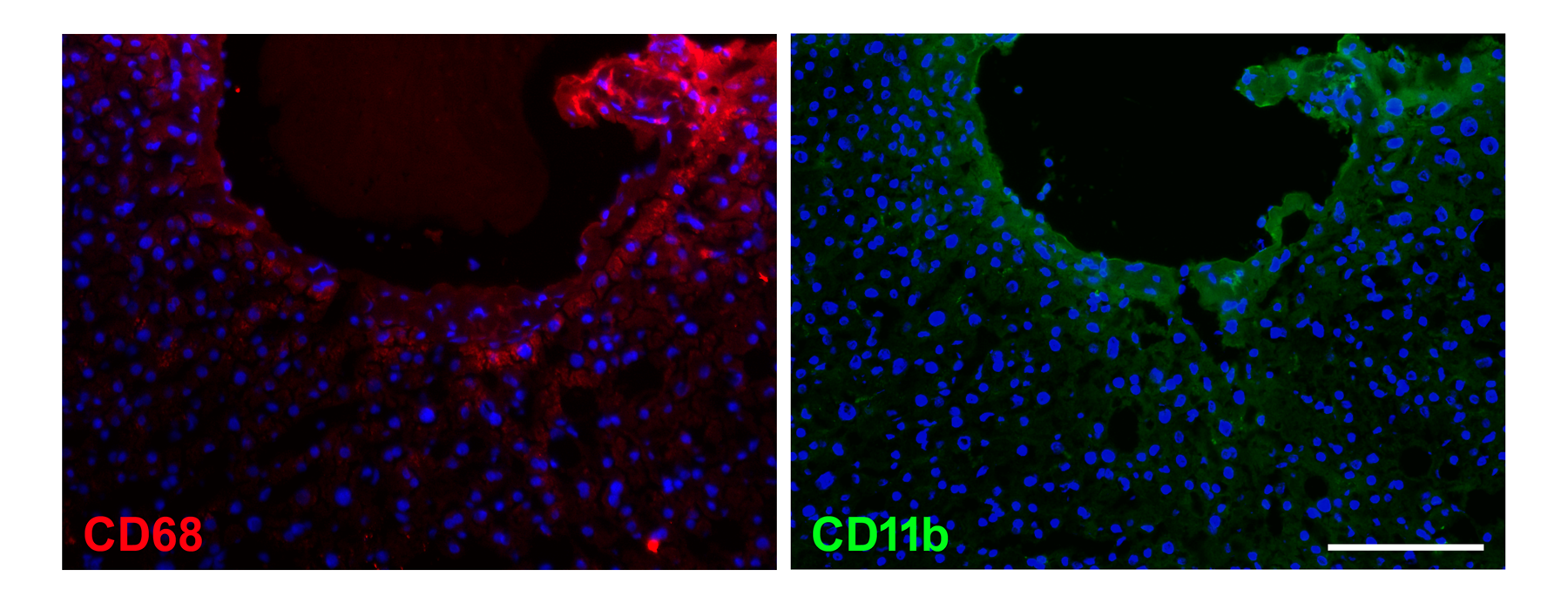

Supplement: Supplementary file 4 — Figure S3. Macrophages in destabilizing atherosclerotic portal venous plaques. IL-1β-related macrophages (marked by CD68) were mainly CD11b (−), suggesting an origin from Kupffer cells and not from bloodstream monocytes [CD11b (+)]. Scale bar: 100 μm. (TIF 3657 kb) [file 12950_2019_211_MOESM4_ESM.tif]

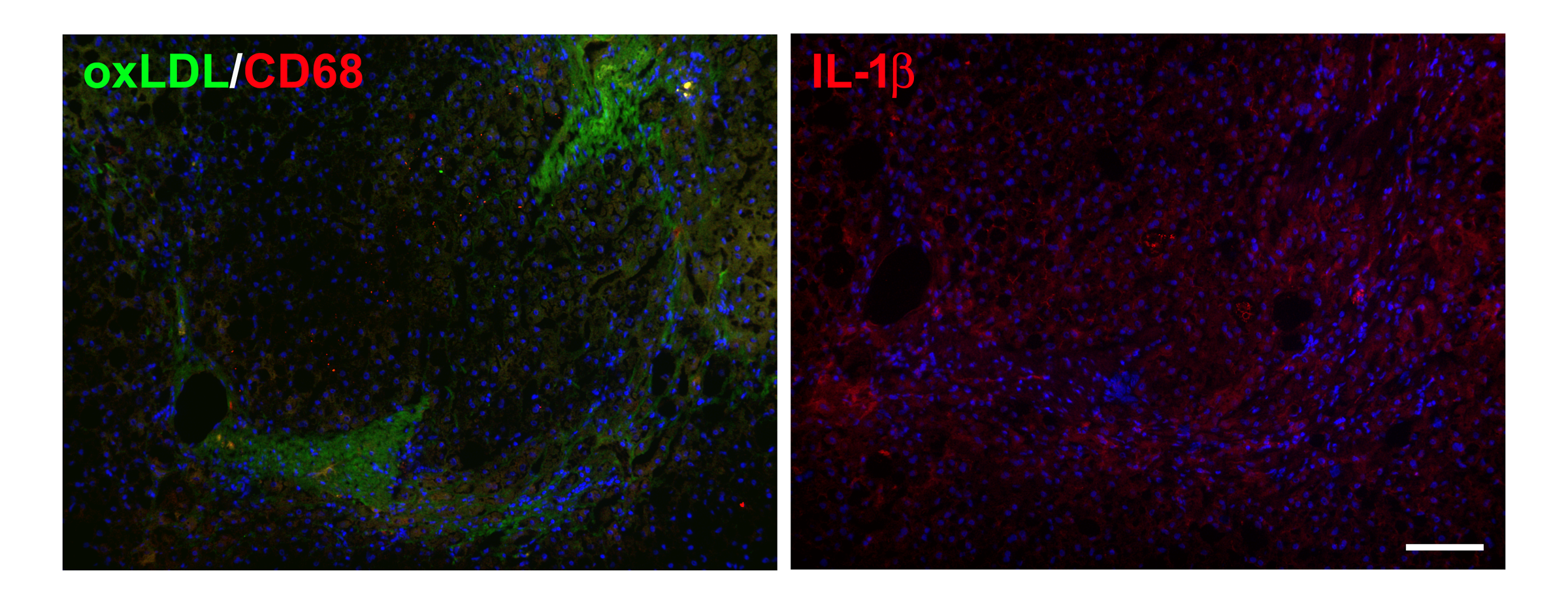

Supplement: Supplementary file 5 — Figure S4. Inactive role of macrophages in fibrotic parenchyma in NAFLD. In the cirrhotic stage, oxLDL was observed in the fibrotic parenchyma. Macrophages (marked by CD68) were rarely visible. IL-1β expression was also low. Scale bar: 100 μm. (TIF 6498 kb) [file 12950_2019_211_MOESM5_ESM.tif]

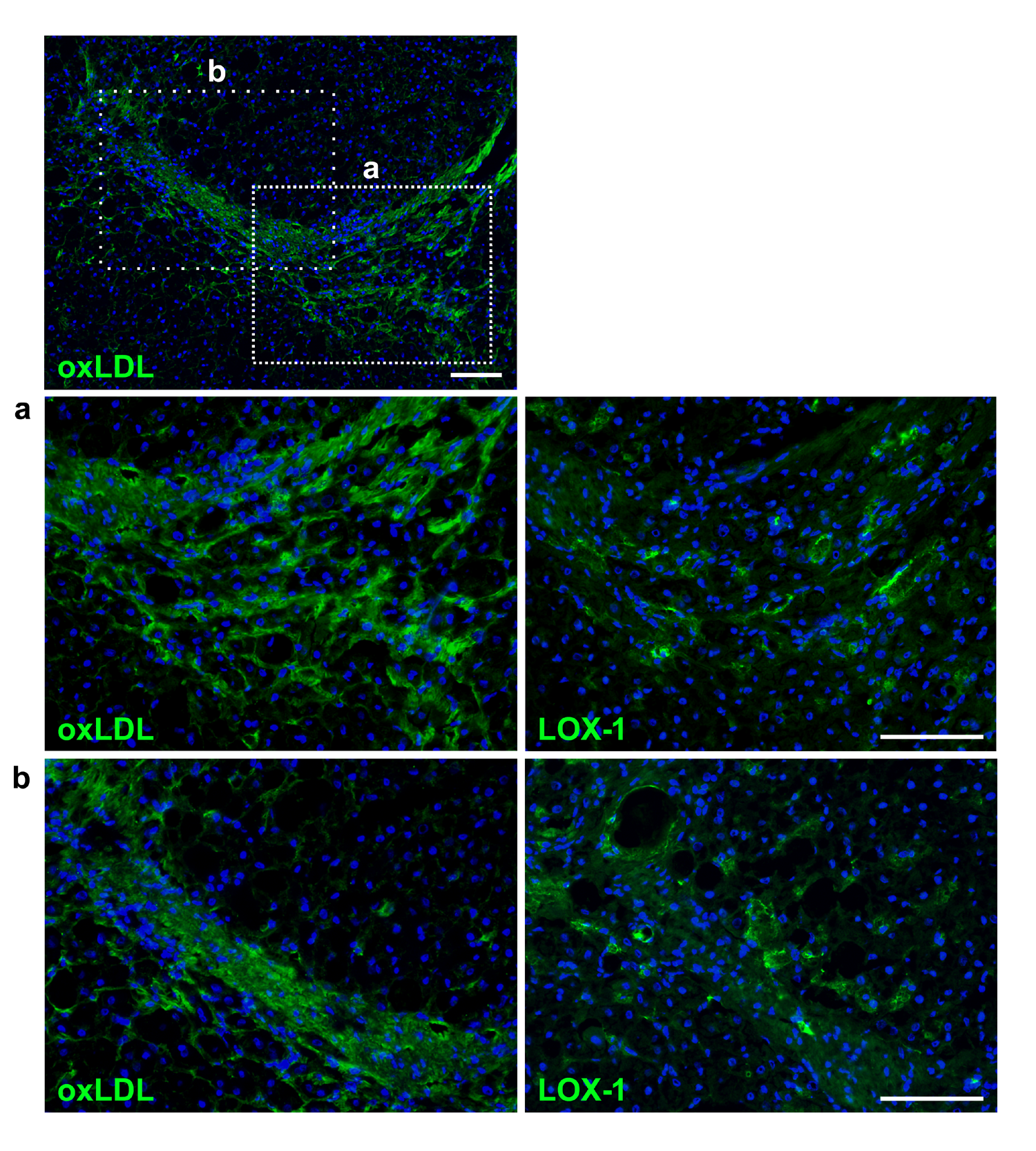

Supplement: Supplementary file 6 — Figure S5. High-power field view of chicken-wire fibrosis in the cirrhotic stage of NAFLD. oxLDL accumulation in chicken-wire fibrosis in the parenchyma distal (a) and adjacent (b) to the portal vein. LOX-1 expression was low in the fibrotic parenchyma and was not closely related to oxLDL accumulation. Scale bar: 100 μm. (TIF 7340 kb) [file 12950_2019_211_MOESM6_ESM.tif]
