# Supplementary material for: Accumulation of free cholesterol and oxidized low-density lipoprotein is associated with portal inflammation and fibrosis in nonalcoholic fatty liver disease
Source: J Inflamm (Lond). 2019 Apr 2;16:7. doi: 10.1186/s12950-019-0211-5 (PMC6444889; doi:10.1186/s12950-019-0211-5)
Supplement: Supplementary file 7 — Supplemental Results. (DOCX 15 kb) [file 12950_2019_211_MOESM7_ESM.docx]

**Supplemental Results**

**Patient Demographics**

Control: Liver (Panel I, Figure S1) was from a 1-year-5-month-old boy with propionic acidemia. He had undergone a liver transplant with a graft from his mother, a living donor. His serum aspartate aminotransferase (AST), alanine aminotransferase (ALT), γ-glutamyl transferase (GGT) and ammonia before liver transplantation were 38 U/L, 25U/L, 12 U/L, and 75 μmol/L, respectively. No liver pathology was diagnosed.

Fatty liver (Panel II, Figure S1) was from a 28-year-old living liver donor to his daughter and he was diagnosed with 5-10% fatty liver in histology. His serum AST, ALT, and GGT, before donor operation were 24 U/L, 32 U/L, and 42 U/L; blood levels of total cholesterol, triglyceride and fasting glucose were 238 mg/dL, 95 mg/dL, and 84 mg/dL, respectively. Body mass index (BMI) was 26.1.

NASH: Liver (Figure 1) was from an 82-year-old man (BMI, 23.1) with underlying 1-vessel coronary arterial disease and hypertension. A 14-cm hepatocellular carcinoma (HCC) was found on the right lobe of the patient’s liver and was resected uneventfully. Serum AST, ALT, GGT and fasting glucose before the operation were 40 U/L, 97 U/I, 107 U/L, and 106 mg/dL, respectively. Tumor recurrence was noted 1 year after the operation, which was treated with transarterial chemoembolization; further recurrence with additional portal vein thrombosis was treated systemically by sorafenib for 7 months. Cancer progressed slowly two years after the withdrawal of sorafenib due to severe hand-food syndrome.

Cirrhosis: Liver (Figure 3) was from a 75-year-old man (BMI, 29.8) with a history of type 2 DM, hyperlipidemia, and moderately fatty liver. His serum AST, ALT, and GGT, before the operation were 33 U/L, 38 U/L, and 26 U/L; blood levels of total cholesterol, LDL, triglyceride and fasting glucose were 159 mg/dL, 103 mg/dL, 209 mg/dL, and 125 mg/dL, respectively. He received central hepatectomy for pathologically diagnosed combined hepatocellular- and cholangio-carcinoma of 3 cm in size. Cancer recurrence was noted 2 years after surgery and was treated with radiofrequency ablation
